# Structural investigations of the α_12_ Si–Ge superstructure

**DOI:** 10.1107/S1600576715000849

**Published:** 2015-01-30

**Authors:** Tanja Etzelstorfer, Mohammad Reza Ahmadpor Monazam, Stefano Cecchi, Dominik Kriegner, Daniel Chrastina, Eleonora Gatti, Emanuele Grilli, Nils Rosemann, Sangam Chatterjee, Vaclav Holý, Fabio Pezzoli, Giovanni Isella, Julian Stangl

**Affiliations:** a Institute of Semiconductor and Solid State Physics, Johannes Kepler University Linz, Altenbergerstrasse 69, A-4040 Linz, Austria; b Zentrum für Oberflächen- und Nanoanalytik, Johannes Kepler University Linz, Altenbergerstrasse 69, A-4040 Linz, Austria; c L-NESS, Dipartimento di Fisica, Politecnico di Milano, Polo di Como, via Anzani 42, I-22100 Como, Italy; d Charles University, Department of Condensed Matter Physics, Ke Karlovu 5, CZ-121 16 Prague 2, Czech Republic; e L-NESS and Dipartimento di Scienza dei Materiali, Universitá degli Studi di Milano-Bicocca, via Roberto Cozzi 55, I-20125 Milano, Italy; f Philipps-Universität Marburg, Fachbereich Physik, AG Experimentelle Halbleiterphysik, Renthof 5, D-35032 Marburg, Germany

**Keywords:** X-ray diffraction, silicon wafers, direct bandgap materials, superlattice structure

## Abstract

This article^1^ reports the X-ray diffraction-based structural characterization of the α_12_ multilayer structure SiGe_2_Si_2_Ge_2_SiGe_12_ [d’Avezac, Luo, Chanier & Zunger (2012 ▶). *Phys. Rev. Lett.* **108**, 027401], which is predicted to form a direct bandgap material. In particular, structural parameters of the superlattice such as thickness and composition as well as interface properties, are obtained. Moreover, it is found that Ge subsequently segregates into layers. These findings are used as input parameters for band structure calculations. It is shown that the direct bandgap properties depend very sensitively on deviations from the nominal structure, and only almost perfect structures can actually yield a direct bandgap. Photoluminescence emission possibly stemming from the superlattice structure is observed.

## Abstract 2

X-ray diffraction-based structural analysis results of possible direct bandgap Si/Ge superlattices composed of monolayer thin deposits are presented, together with theoretical predictions and first optical measurements.

## 1. Introduction   

The integration of optical and electronic functions on a silicon wafer would greatly enhance the capabilities of CMOS-based devices; however, the indirect bandgap of Si is a major constraint for the achievement of efficient light emission/detection. Ge, an indirect bandgap semiconductor, highly compatible with Si technology, has been predicted to exhibit a direct gap under high tensile strain (Zhang & Crespi, 2009 ▶), and various fabrication approaches have been proposed (Sun *et al.*, 2009 ▶; Süess *et al.*, 2013 ▶; Sukhdeo *et al.*, 2014 ▶).

Other methods to achieve the monolithic integration of an efficient light source into Si, based on band structure manipulation, are nanostructuring into nanowires (Nolan *et al.*, 2007 ▶) or quantum dots, or alloying with Sn (Moontragoon & Harrison, 2007 ▶).

The combination of nanostructuring, alloying and strain engineering has been discussed for almost 30 years (Kasper & Theodorou, 2007 ▶). The zone-folding approach has been used to identify an Si
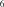
Ge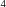 superlattice (SL),^2^ when grown under (001) epitaxial strain, which exhibits a small, but nonzero, absorption at the band edges due to a nearly direct bandgap (Froyen *et al.*, 1987 ▶; Pearsall *et al.*, 1989 ▶).

Since the available computational power has grown considerably in the past few decades and novel methods have been developed, the search to identify more complex motifs can now be carried out. d’Avezac *et al.* (2012 ▶) used genetic algorithms and band structure calculations based on density functional theory (DFT) to search within the pool of all possible Si and Ge monolayer stacking sequences ([Si
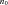
Ge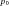/Si
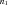
Ge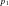/…/Si
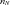
Ge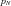]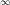) epitaxically grown on different substrate orientations and with different strain levels in order to identify sequences with strong direct transitions across the electronic bandgap. Indeed, such motifs have been found, and surprisingly, all are variants of a magic sequence [SiGe
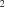
Si
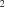
Ge
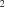
Si] sandwiched between a 12–32 monolayer-thick Ge spacer layer deposited on an Si
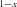
Ge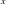 virtual substrate with 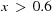.

d’Avezac *et al.* (2012 ▶) also discussed the robustness of the transitions upon deviations from the ideal structure. It has been found that the transitions are quite robust against variations of the Ge content in the spacer layer. The directness is not influenced if this spacer layer is not pure Ge as long as it is Ge rich. What influences the transition strength most is the structural precision of the layers within the magic sequence in terms of interface mixing. It has been found that the transition strength remains more than 65% of the original value up to an intermixing of 10%, *i.e.* Si
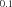
Ge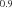 instead of Ge and Si
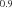
Ge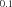 instead of Si. Thus, such a high structural quality is eligible but experimentally difficult to achieve. For optically active SiGe structures, a low density of defects such as dislocations is also important but difficult to obtain when Ge-rich slabs have to be grown on Si. Thick relaxed Si
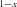
Ge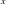 virtual substrates have been demonstrated to be one of the most effective approaches in reducing the threading dislocation density in the active layers (Fitzgerald *et al.*, 1991 ▶). In order to avoid additional dislocation nucleation in the optically active material, the SLs have to be deposited in a strain symmetrized fashion, which implies that the SL’s average Ge content matches that in the buffer underneath and the constituting Ge and Si layers are under compressive and tensile strain, respectively. The structure with the lowest Ge content and therefore the thinnest possible virtual substrate under these constraints of strain symmetrization is the 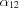 structure (magic motif sandwiched between 12 monolayers of Ge) which features, on average, an 80% Ge content.

The growth of the magic motif along with photoluminescence (PL) studies has only been reported on Si
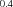
Ge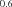 buffers (Lockwood *et al.*, 2014 ▶).

In this work the growth of the 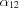 structure in a thick SL is reported, and a detailed structural investigation of the achieved structural quality is presented, together with first PL results and band structure calculations including Si–Ge intermixing.

### 1.1. Sample preparation   

**Figure 1:**
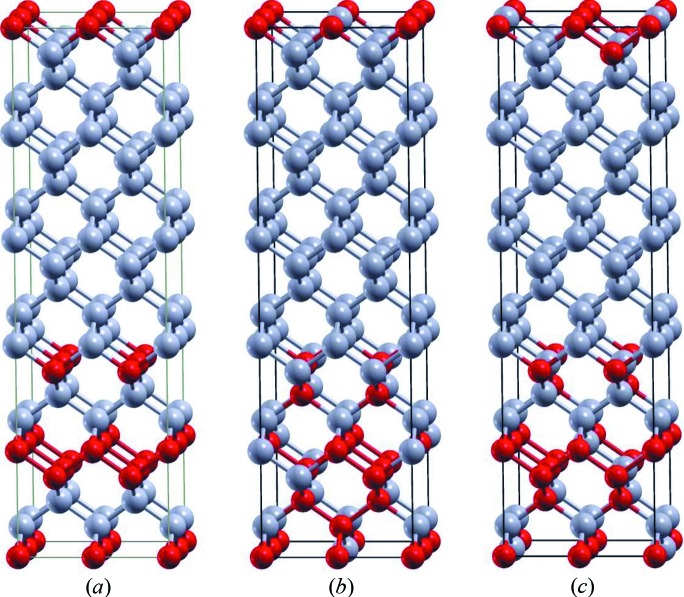
Panel (*a*) shows the magic motif SiGe
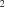
Si
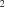
Ge
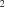
Si together with the 12 monolayers of Ge constituting the layer sequence of the 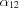 structure. Panels (*b*) and (*c*) show two different configurations of the magic motif together with the Ge spacer layer including segregation effects.

The 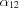 structure, as depicted in Fig. 1 ▶(*a*), was grown on an Si
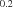
Ge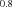 virtual substrate to achieve strain compensation and avoid plastic relaxation in the SL. Linearly graded virtual substrates exhibit a relatively low threading dislocation density (approximately 10
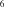
 cm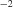); however, in order to achieve an 80% Ge content 8–12 µm-thick epilayers are required. In such thick epilayers, wafer bending occurs as a mechanism to relax the thermal tensile strain developed during the cool-down phase after deposition, whereas for accurate X-ray analysis, especially X-ray reflectivity measurements, flat samples are needed. A double-step graded thin buffer, which guarantees an r.m.s. roughness below 1 nm and prevents wafer bending, has therefore been used (Cecchi *et al.*, 2014 ▶). The whole structure including both the buffer and the magic motif was grown without interruption on RCA-cleaned HF-dipped Si(001) substrates (*p*-type, 1–10 Ωcm) by low-energy plasma-enhanced chemical vapor deposition (Isella *et al.*, 2004 ▶).

The theoretical calculations all assume periodic boundary conditions, *i.e.* an infinite number of periods. In practice, the first and final few SL periods will show different properties from the middle layers owing to dissimilar start conditions and surface oxidation. In order to obtain enough active material, rather thick SL stacks need to be fabricated. Thus three SL samples with 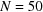, 20 and 10 periods have been grown. The 50 period sample provides sufficient material for optical applications and is expected to approach the bulk behavior with respect to its optical properties. Although period fluctuations and drifts are inevitable during growth, SLs with a lower number of periods are expected to exhibit a higher structural quality. Fitting of all the structural parameters (layer composition, layer thickness, interface roughness, and lateral and vertical correlation length, as described below) of an SL with the eight different monolayers of the magic motif plus the Ge spacer monolayers already gives a very large number of fitting parameters without taking into account growth fluctuations. Therefore, the sample with 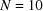 periods is intended for structural analysis, where growth fluctuations can be neglected. Drifts and composition fluctuations are then introduced for the 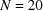 and 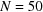 period samples.

## 2. X-ray scattering and structural analysis   

**Figure 2:**
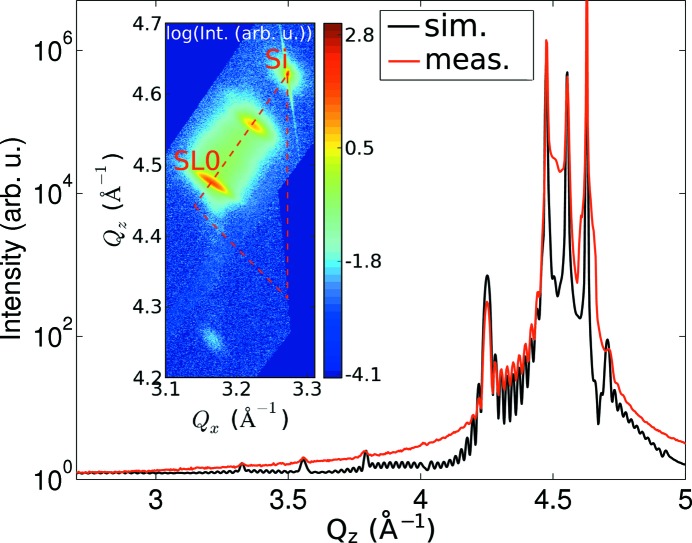
Integrated intensity line plot along 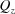 of the symmetric RSM around the 004 Si Bragg peak, together with the Takagi–Taupin fit for the 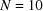 period sample (integration along 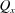). The inset shows the asymmetric RSM around the 224 Si Bragg peak together with the SiGe relaxation triangle. The position of the Si substrate peak, as well as the zeroth-order SL peak (SL0) which coincides with the second step of the virtual substrate, is labeled. The coherent growth of the superlattice on the two-step graded buffer with final Ge content of 82.7 (5)% is confirmed. In the simulation the grading from the first to the second step of the virtual substrate has not been included for simplicity.

**Figure 3:**
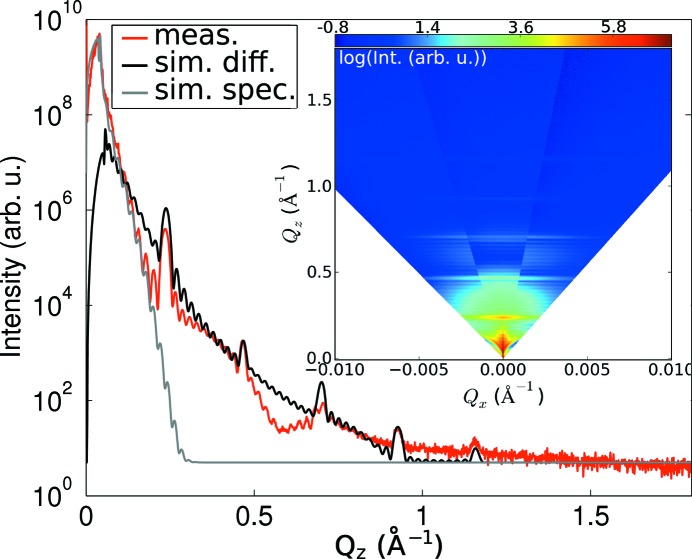
Line cut along 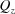 of the RSM around the 000 Bragg reflection, together with the fitted specular and diffuse scattering signal for the 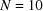 period sample. The inset shows the RSM around the origin of reciprocal space.

At beamline P08 at PETRA III in Hamburg, symmetric X-ray diffraction (XRD) and X-ray reflectivity (XRR) reciprocal space maps (RSMs) in coplanar scattering geometry were obtained with an energy of 10.5 keV for all three samples. Asymmetric RSMs were obtained with a custom built laboratory rotating anode setup using Cu *K* 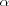 radiation. In Fig. 2 ▶ the integrated intensity (integration along 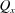) curve along 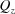 of the symmetric RSM around the 004 Si Bragg peak is shown, together with the recorded asymmetric RSM around the 224 Si Bragg peak for the sample with *N* = 10 periods. Fig. 3 ▶ shows the line cut at 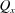 = 0 of the XRR map around the 000 Bragg reflection, together with the full map in the inset.

In the asymmetric RSM (inset of Fig. 2 ▶) it can be seen that the SL is strain symmetrized with respect to the virtual substrate. Indeed, the 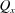 position of the zeroth-order SL satellite peak (SL0 in the inset of Fig. 2 ▶) corresponds perfectly to that of the constant-composition SiGe top layer of the thin buffer. Thus the strain-compensation condition has been achieved, which means that even for large *N* no further dislocations nucleate. SL peaks are visible up to the fifth order in the integrated intensity line plot of the symmetric RSM (Fig. 2 ▶).

In the reflectivity map (inset of Fig. 3 ▶) it is worth mentioning that many orders of diffuse SL fringes are visible, but the specular rod vanishes for 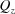 values below 0.2 Å^−1^. This suggests on the one hand a very precise and stable repetition of the layer sequence, which leads to the presence of many SL fringes, but on the other hand a very large roughness which damps this SL peak along the specular path and produces the diffuse SL sheets.

The integrated intensity curves taken from the 004 RSMs were fitted with fully dynamical Takagi–Taupin simulations (Bartels *et al.*, 1986 ▶). With this approach it is possible to determine the thickness, composition and strain of the individual layers. We used a model consisting of the individual monolayers, where the Ge content of each layer was varied. Additionally, a width of the interface region can be given to take into account roughness and interdiffusion. XRD simulations give the same result for either roughness or interdiffusion and hence those two effects cannot be distinguished.

However, from XRR fits it is possible to determine the interface roughness and as a consequence distinguish between roughness and interdiffusion. The existence of resonant diffuse scattering sheets (Pietsch *et al.*, 2004 ▶) extending to finite 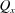 values is connected to the vertical replication of rough interfaces and allows one to determine the ‘true’ interface roughness. The damping of the specular reflectivity yields information on the combined effect of roughness and interdiffusion.

The Parratt formalism (Parrat, 1954 ▶) was used to fit the specular path, whereas for the diffusely scattered intensity a semi-kinematical approach was deployed (Sinha *et al.*, 1988 ▶). Since the diffusely scattered intensity is the Fourier transform of an interface correlation function, typical correlation lengths, both in-plane and in the growth direction, can be determined.

A typical correlation function resulting from the fractal description of random rough surfaces (Holý *et al.*, 1993 ▶) is 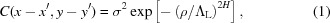
where *x* and *y* are the in-plane coordinates, 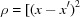
 + 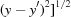, σ is the r.m.s. roughness, 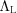 is the lateral correlation length and *H* is the Hurst parameter, which gives information on the jaggedness of an interface: larger *H* values correspond to less jagged interfaces. 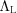 corresponds to the average distance within an interface over which surface features become uncorrelated.

In order to obtain a full picture of the structure, both XRD and XRR (specular and diffuse) signals were fitted using the same set of input parameters.

### 2.1. Results   

In order to match best the intensity ratios of the different superlattice peaks of the XRD signal (shown in Fig. 2 ▶), not only the period had to be varied slightly with respect to the nominal structure, but also the Ge content in the nominally either pure Si or pure Ge layers needed to be adjusted. It has been found that there is always some Ge of the underlying Ge layers present in the subsequent Si layers owing to segregation of Ge, a commonly observed phenomenon (Godbey & Ancona, 1998 ▶). Additionally, to describe the observed damping of the SL peaks in XRD, a very large width of the interface region along the growth direction had to be introduced, which is almost an order of magnitude larger (8–10 monolayers) than the thickness of the thinnest layer. The diffusely scattered intensity in the XRR maps does not vanish up to the fifth-order SL peak; only the specular rod vanishes before the first satellite peak. This can be modeled as locally very well defined but wavy layers. This in turn produces the quickly damped SL peaks in XRD and in the specular XRR, while the satellites in the diffuse intensity in XRR are present up to very high 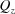 values (see Fig. 3 ▶).^3^ The vertical correlation lengths are longer than the SL thickness, *i.e.* even for the largest observable 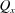 values of the resonant diffuse scattering sheets, their width along 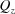 is actually determined by the total SL thickness.

The waviness is very plausible, since the extremely thin layers of the magic motif are grown directly on the virtual substrate, which is already wavy owing to the cross-hatch pattern caused by the misfit dislocations in the two-step buffer. The r.m.s. amplitude of this waviness obtained by atomic force microscopy lies around 1 nm for two-step buffers with final Ge content similar to the used one (Cecchi *et al.*, 2014 ▶). Typical lateral feature sizes correspond well to the lateral correlation lengths of ∼100 nm obtained in the XRR fits.

**Figure 4:**
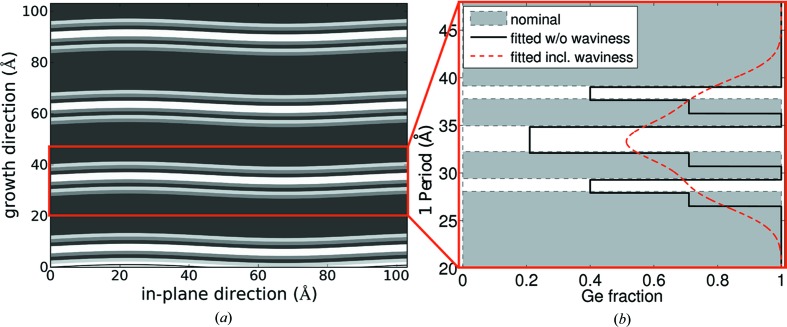
(*a*) A sketch of the obtained structural parameters for the 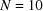 period sample. The gray scale of the layers depicts the Ge content: the darker the more Ge. The exact values are listed in Table 1 ▶. (*b*) The thickness and Ge content of the layers constituting one period. The black curve corresponds to the obtained Ge content depending on the position within one period, while the gray/white background depicts the nominal Ge concentration. The red dashed line shows the smearing of the Ge concentration taking into account the waviness.

The Ge profile obtained by combining XRD and XRR measurements and simulations is shown in Fig. 4 ▶. The waviness, with the lateral frequency found by XRR investigations, is sketched as a sinusoidal variation in Fig. 4 ▶(*a*). In Fig. 4 ▶(*b*) the light-gray/white background in the Ge profile corresponds to the nominal Ge content and the black line denotes the determined Ge content. The red curve includes smearing due to the lateral averaging over the waviness.

These findings, except for slightly different average Ge contents and periods, are very similar for all three samples (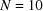, 20 and 50). The detailed results can be found in Table 1 ▶.

In the simulations, different parameters can be determined with different confidence. The position of the SL0 in the XRD maps is extremely sensitive to the average composition of the SL unit cell, which can therefore be determined very accurately and fixed in the simulation process. Similarly, the period length can be determined with very high accuracy and is kept fixed. The composition of the individual layers of the magic motif, which is ‘encoded’ in the relative intensities of the satellite peaks in XRD and XRR, can be determined with less confidence. Changing only the composition of a single layer, and compensating for the according changes in average composition and period by slightly modifying the spacer layer, is possible within about 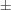
15%. (Since our model consists of monolayers, the thickness of each of them is determined from an interpolation between the thicknesses of a pure Si or pure Ge monolayer, considering the tetragonal distortions due to the strain in each layer.) However, bearing in mind that any segregation or interdiffusion will be rather similar in the individual layers of the magic sequence, and thus linking the compositions of the (nominally pure) Si and Ge monolayers accordingly, results in confidence intervals of about 10% for the compositions of those layers. These ‘reasonable’ confidence intervals are given in Table 1 ▶.

Comparing the structural results with the range of beneficial parameters for direct bandgap formation obtained by d’Avezac and co-workers, which require an intermixing below 10% (d’Avezac *et al.*, 2012 ▶), the interdiffusion is quite considerable, especially concerning the Si layers. The large waviness is not very critical, since locally the SL structure remains intact. The individual layer thicknesses in the structures match the 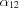 motif rather well. Thus it is not clear beforehand whether such structures can be expected to exhibit a direct bandgap or not. Therefore, PL investigations and theoretical band structure calculations have been performed.

## 3. Optical properties: theory and experiment   

Performing theoretical simulations of the band structure of the wavy and interdiffused multilayer stack described above is virtually impossible, even with today’s computational power. Therefore, a simplified approach has been followed to capture the main experimental findings, while keeping the resulting unit cells as small as possible. The waviness occurs on comparatively large length scales and does not modify the local layer structure significantly; hence it has been ignored in the simulations. Instead, calculations have been focused on the effect of segregation on electronic and optical properties. For this purpose, it has been considered that Ge segregates to the top during SL growth, so if an Si layer is added, half the Si atoms exchange places with nearest neighbor atoms in the layer below (*i.e.* a single Si layer on top of pure Ge transforms into two Si
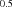
Ge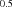 layers), but if a Ge layer is deposited, the Ge atoms stay in the top layer. In this way, the nominal sequence of Ge/Ge/Si/Ge transforms into Ge/Si
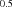
Ge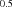/Si
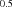
Ge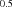/Ge, and a sequence Ge/Ge/Si/Si/Ge transforms to Ge/Si
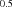
Ge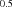/Si
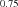
Ge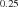/Si
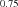
Ge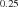/Ge *etc*. While this approach is rather coarse (the smallest possible change in composition is 25%), it qualitatively captures the observed Ge profiles in the SL stack. Actually, it overestimates the segregation in order to determine the trends in the band structure upon intermixing. Limiting the jumps in chemical composition to 25% allows us to perform the calculations with a unit cell only four times larger in-plane than the one used for the ideal sequence. It has been also considered that the first Si layer of the sequence is intermixed with Ge from the underlying Ge spacer layer, implying that actually none of the Si layers are intact and pure. For a certain quantity of exchanged Si and Ge atoms in a layer, several possible representations in the 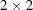 unit cell of the calculation exist. Two different ones are shown in Figs. 1 ▶(*b*) and 1 ▶(*c*), where the gray (light) and red (dark) circles represent Ge and Si atoms, respectively. Fig. 1 ▶(*a*) shows the ideal 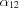 sequence for comparison.

To obtain the band structure for the different interdiffused sequences, the scheme applied to the ideal 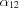 sequence by Monazam *et al.* (2013 ▶) was used: the local density approximation (LDA) in the framework of density functional theory (DFT) was deployed for exchange-correlation effects. The plane-wave code *ABINIT* (Gonze *et al.*, 2002 ▶; Gonze, 2005 ▶), together with norm-conserving Troullier–Martins scheme pseudopotentials (Troullier & Martins, 1991 ▶), was used for LDA/DFT calculations. For all calculations the same relative density *k*-mesh as for 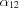 was employed to obtain electronic properties. Additional parameters required for total energy convergence were kept the same as in the work of Monazam *et al.* (2013 ▶).

**Figure 5:**
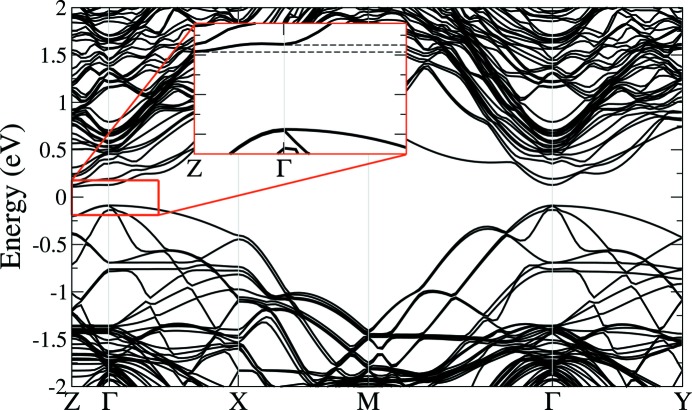
Calculated band structure for the sequence with partly intermixed layers as depicted in Fig. 1 ▶(*b*). The trends are the same for the structures in Figs. 1 ▶(*b*) and 1 ▶(*c*): the fundamental bandgap is lowered compared to the fundamental bandgap of the ideal 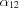, the indirect bandgap shifts about 10 meV below the direct one (dashed lines in close-up inset).

The band structures of both considered intermixed SLs show the same trends; that for the structure in Fig. 1 ▶(*b*) is depicted in Fig. 5 ▶. The most important feature in both cases is that the band structure is not direct anymore. The minimum of the conduction band is now located between the Γ and *Z* points in reciprocal space and not at the Γ point as in the 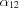 structure (see inset of Fig. 5 ▶ for details). These bands appear as a result of zone back-folding of bands from the 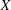
–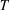 line to the 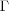
–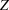 line in the Brillouin zone, due to doubling of the unit-cell size in the *a* and *b* directions [the nomenclature of high-symmetry points used here is described, for example, by Schmid *et al.* (1991 ▶)].

Another important difference found from the LDA calculation is a reduction of the fundamental bandgap by about 70 meV with respect to the 0.61 eV of the ideal 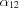 structure (Monazam *et al.*, 2013 ▶). The direct bandgap in the Γ point is only about 10 meV larger than the indirect one. Considering the usual underestimation of bandgap values in DFT/LDA calculation, and renormalizing the values with GW calculations of the ideal 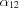 structure (the GW approximation is the many-body perturbation theory for describing the quasi-particles; Hedin, 1965 ▶), an actual value of around 0.55 eV for the fundamental gap of the interdiffused structure can be estimated.

**Figure 6:**
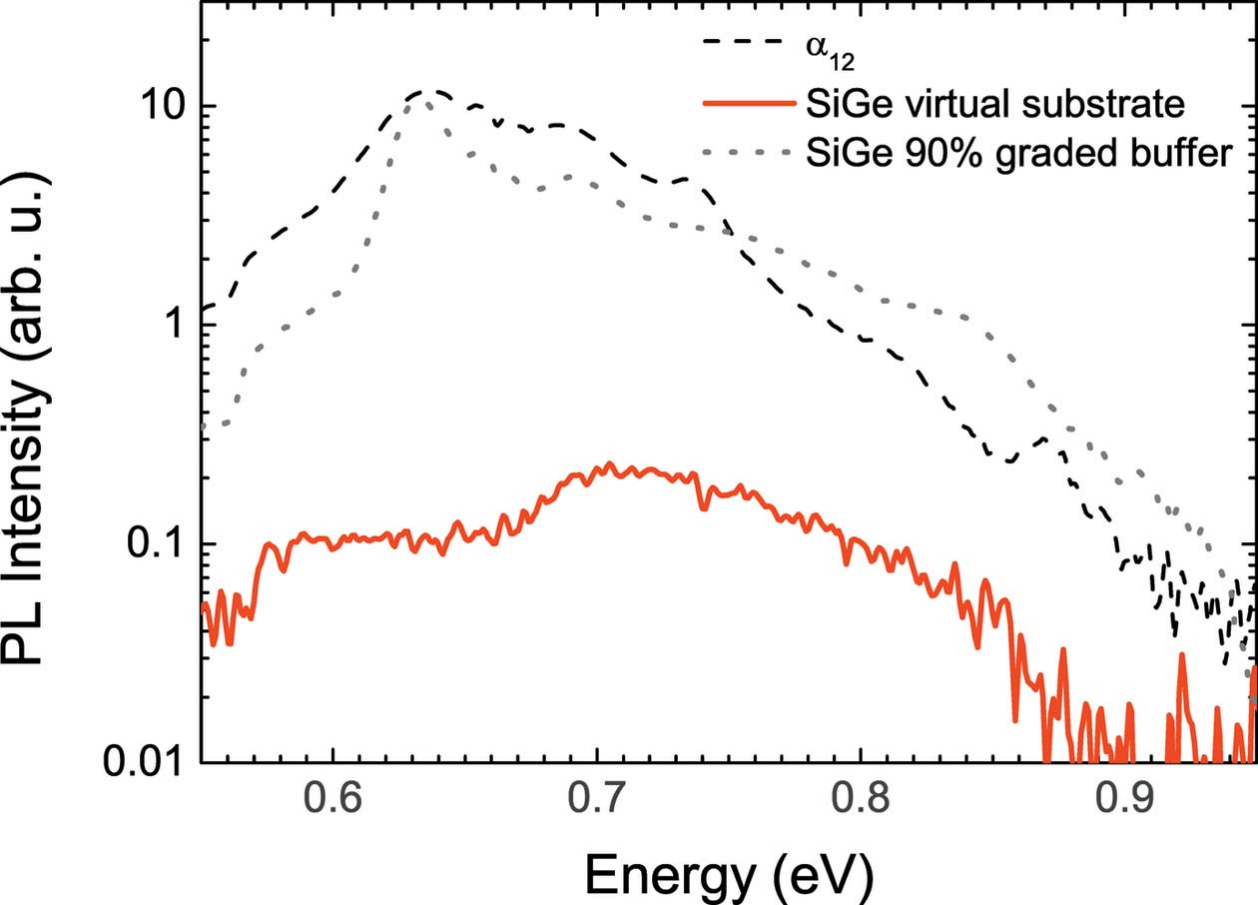
Preliminary photoluminescence results of the 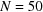 period sample as grown together with the result of the virtual substrate. It can clearly be seen that the majority of the signal is attributed to the SL structure. The found peak is very close to the predicted fundamental bandgap of 0.61 eV (Monazam *et al.*, 2013 ▶). Nevertheless, defects could not be completely eliminated as the source of the PL of the SL structure, as discussed in the text.

Despite not matching the nominal structure perfectly, preliminary PL measurements on the 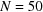 period sample show a prominent emission feature around 0.63–0.64 eV, as depicted in Fig. 6 ▶ (black dashed line), which is close to the fundamental bandgap of 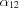 predicted by Monazam *et al.* (2013 ▶). A tail of nonzero PL intensities for slightly lower transition energies is also observed. In order to exclude defects as the source of the optical emission, PL measurements on the two-step virtual buffer have also been carried out, and show virtually no signal in this region (solid red line in Fig. 6 ▶). Since the number of dislocations and their nature does not change upon the SL growth on the virtual substrate, the comparison of those two signals shows that the emission is possibly related to the superlattice. Nevertheless, the emission of defects in the Ge-rich alloyed superlattice cannot be completely ruled out. Standard Si
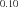
Ge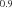 alloy layers grown on graded buffers show PL features in the same energy range as the 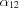 structure (gray dotted line in Fig. 6 ▶).

In order to study the nature of the emission signal in more detail the SL was intentionally destroyed and the PL measurements were repeated. For this experiment, three nominally identical pieces of the 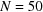 period sample were annealed at different temperatures while the specular XRR signal was monitored *in situ*. It was observed that after annealing for 2 h at 873 K the magic motif was interdiffused, while this interdiffused part together with the Ge spacer layer still formed a superlattice. By annealing for 2 h at 923 K, the magic motif interdiffused also with the Ge spacer layer, but still some modulation of Ge content within one SL period was present, leading to SL peaks in XRR. After 2 h at 1023 K the SL structure had been completely alloyed, resulting in a complete extinction of the SL peaks.

Preliminary PL data of those interdiffused samples show clear differences compared to the as-grown sample. For a detailed understanding of the observed PL lines and a correct identification of the 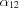 features, however, additional optical investigations together with more detailed theoretical calculations of the band structure and transition matrix elements have to be carried out. In particular, Ge profiles better matching the obtained ones need to be implemented, which requires larger unit cells. The PL data together with the final theory results will be reported elsewhere.

## 4. Conclusions   

In conclusion, detailed structural investigations of actual magic motif SLs have been performed and the parameters for interdiffusion and interface roughness, chemical composition in all individual layers, and the layer thicknesses have been obtained. The results have been used for band structure calculations, showing that such deviations from the nominal structure are prone to change the nature of the fundamental gap, which becomes indirect. Nevertheless, PL emission in the region of the predicted direct bandgap has been observed. While this is rather promising, further optical and growth studies are needed to finally clarify the details of the observed PL signals.

## Figures and Tables

**Table:** In the first part of the table, general parameters of the three samples are given, namely the period (*p*), the average Ge content (Av. 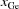), the period fluctuations (standard deviation 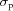), the Hurst parameter *H*, and the lateral and vertical correlation lengths (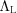 and 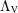, respectively). The following part gives parameters of the individual monolayers, namely the thickness (*t*), the Ge fraction (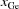, a value between 0.0 and 1.0) and the amplitude of the waviness (). The thick Ge spacer layer has been handled as two individual layers in order to treat the region adjacent the magic motif differently. To keep the number of fit parameters reasonable, single and double monolayers were treated identically. Values in parentheses are errors on the least significant digits.

Sample	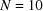			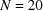			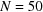		
*p* ()	27.92 (10)			27.27 (10)			27.05 (10)		
Av. 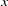 _Ge_	0.827 (5)			0.824 (5)			0.827 (5)		
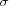 _p_ ()	0.0 (5)			0.0 (5)			0.0 (5)		
*H*	0.5			0.5			0.5		
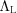 ()	900 (300)			900 (300)			900 (300)		
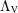 ()	600 (200)			600 (200)			600 (200)		

**Table:** 

Sample	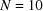			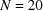			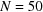		
	*t* ()	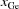	()	t ()	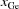	()	*t* ()	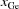	()
	(10)	(10)	(5)	(10)	(10)	(5)	(10)	(10)	(5)
Si	1.38	0.40	12	1.38	0.40	11	1.38	0.40	12
Ge	1.41	1.00	9	1.41	1.00	8	1.41	1.00	9
Ge	1.40	0.71	10	1.40	0.73	9	1.40	0.77	10
Si	1.37	0.21	11	1.37	0.25	10	1.37	0.25	11
Si	1.37	0.21	12	1.37	0.25	11	1.37	0.25	12
Ge	1.41	1.00	9	1.41	1.00	8	1.41	1.00	9
Ge	1.40	0.71	10	1.40	0.73	9	1.40	0.77	10
Si	1.38	0.40	12	1.38	0.40	11	1.38	0.40	12
Ge	15.35	1.00	9	14.74	1.00	8	14.51	1.00	9
Ge	1.40	0.71	10	1.40	0.73	9	1.40	0.77	10

## Footnotes

2 Integer subscripts correspond to numbers of monolayers, whereas decimal number subscripts denote concentrations.

3 In the 004 line scan measured at , only the laterally averaged interface structure is observed.
